# Co-spraying of carriers (mannitol-lactose) as a method to improve aerosolization performance of salbutamol sulfate dry powder inhaler

**DOI:** 10.1007/s13346-020-00707-6

**Published:** 2020-01-13

**Authors:** Mickey Socrates Ferdynand, Ali Nokhodchi

**Affiliations:** 1 Pharmaceutics Research Laboratory, School of Life Sciences, University of Sussex, Brighton, BN1 9QJ UK; 2 Drug Applied Research Center and Faculty of Pharmacy, Tabriz University of Medical Sciences, Tabriz, Iran

**Keywords:** Spray-dried carriers, Leucine, Inhalation performance, Dry powder inhaler, Salbutamol sulfate, Polymorphism, Ternary mixtures

## Abstract

Although in dry powder inhaler (DPI) formulations a single carrier is used, a single carrier is not able to provide an excellent aerosolization performance when it is used in DPI formulations. Thereby, the aim of this study was to engineer a suitable ternary mixture of mannitol-lactose-leucine to be used in a DPI formulation with enhanced aerosolization performance. To this end, binary mixtures of mannitol:lactose containing a constant amount of leucine (5% w/w of carriers) were spray-dried as a single solution. Spray-dried samples were blended with salbutamol sulfate to determine the efficiency of their aerosolization performance. Interestingly, note that lactose was in its amorphous state stabilized by the presence of mannitol in the samples. Spray-dried mannitol without lactose showed a combination of the α- and β-polymorphic forms which was the case in all other ratios of mannitol:lactose. It was shown that the highest fine particle fraction (FPF) was 62.42 ± 4.21% which was obtained for the distinct binary mixtures (1:3 mannitol:lactose) compared to a single carrier. This study opens a new window to investigate further the implementation of binary mixtures of sugar carriers containing leucine in DPI formulations to overcome poor aerosolization performance the mentioned DPI formulations.

## Introduction

β2-Adrenergic receptor agonists, such as salbutamol sulfate, act on the β2-adrenergic receptor to facilitate smooth muscle relaxation and dilation of bronchial passages [1]. This enables patients suffering from a disease like that of asthma, which is a chronic inflammatory disease characterized by the obstruction of airflow due to bronchial airway constriction in response to a stimulus [2], the ability to breathe. Such process, however, is achieved via the delivery of a successful therapeutic dosage of salbutamol sulfate to be deposited more peripherally (in the middle and small airway) [3, 4]. Therefore, aerosolization performance becomes a significant factor in the development and implementation of any given dry powder inhaler (DPI) formulation given that inter-particulate forces are directly influenced by particle density, morphology, surface roughness, particle-size distribution, presence of fine particle excipients, surface energy, carrier flow, and the type of carrier chosen [5, 6].

It has been shown that spray drying can be used to manipulate the properties of a carrier to achieve a better aerosolization performance through aggregated spray-dried lactose particles [7]. Nevertheless, the array by which spray drying becomes applicable coincides with its advantages over other traditional methods such as ball milling or micronization. Although there are other emerging technologies such as microfluidics [8], electrohydrodynamic atomization (EHDA) have also been introduced [9], and the spray drying method is currently the main process used to produce particles.

The introduction of leucine has been documented to provide advantages to DPI formulations [10] while also enhancing both powder dispersion [11–14] protecting against moisture [15, 16] eluding a lubrication effect [7] and providing increased stability for the formulation via hydrogen bonding and van der Waals forces. In addition, its use as a co-processed excipient has also been shown to stabilize DPI formulations containing highly hygroscopic carriers such as lactose, while maintaining a consistent inhalation performance after 6 months [11].

It has been shown that leucine is a surfactant and has both hydrophobic and hydrophilic groups. Leucine enriches the particle surface during the spray drying and could generate hydrophobic surfaces [17]. It has been shown that changes in the hydrophilicity of carriers play an important role in moisture uptake process and make DPI formulation less hygroscopic [18]. This, in turn, results in less cohesive particles and a possible decrease in particle size due to the surfactant behavior of leucine, reducing the size of droplets produced during atomization [19], hence a better deposition.

Lactose and mannitol account for two of the most commonly used inactive pharmaceutical ingredients, with the former being the most common, for purposes of drug delivery and, the latter, being labeled as a good alternative to lactose in DPI formulations [20, 21]. Nevertheless, their chemical properties vary significantly with lactose being considered a reducing sugar while mannitol lacks that capability given the absence of the carbonyl group, present in lactose. Their unique chemical profiles allow for them to be labeled as generally recognized as safe (GRAS) by the US Food and Drug Administration (US FDA) and by the European Pharmaceutical Union (EPU). It has been shown that the incorporation of leucine in spray-dried lactose [7] or mannitol [22] could improve the aerosolization performance of salbutamol sulfate, and this was the case when binary mixtures of mannitol-lactose were used [23, 24]. However, none of the previous studies employed spray drying technology to spray dry binary mixtures of sugars containing leucine simultaneously (ternary mixtures overall). In the previous studies performed by Kaialy et al. [23] and Yang et al. [24], only API was spray-dried and physically mixed with carriers. There is no study on the co-spraying of these sugars in the presence of leucine for improved aerosolization of salbutamol sulfate in DPI formulations. Therefore, the aim of this study was to invent a DPI formulation suitable for salbutamol sulfate containing co-spray-dried sugars with leucine to determine its efficacy and potential use as an engineered carrier. Furthermore, the use of various ratios of mannitol:lactose can tailor the binary mixture to be suitable as a carrier for DPI formulations.

## Materials and methods

### Materials

Mannitol was supplied from Roquette (Lestrem, France; purity 99.9%), salbutamol sulfate from L.B. (Bohle, Germany; purity, 99.9%), lactose from DFE Pharma (Gosh, Germany; purity, 99.9%), and L-leucine (purity, 99.9%) and the monobasic potassium phosphate by Acros Organics (Geel, Belgium; purity, 99.9%). All solvents (methanol, ethanol, and hydrochloric acid) were purchased from VWR International Ltd. (Leighton Buzzard, UK) and were high-performance liquid chromatography (HPLC) grade. Fisher Scientific™ (UK) provided the 0.22-μm filters.

### Spray drying

Spray drying was conducted using the Mini Spray Dryer B-290 from Buchi (Flawil, Switzerland) equipped with a dehumidifier (Dehumidifier B-296), an inert loop (Inert Loop B-295), and an outlet filter in a closed system with the use of nitrogen (N_2_) gas. Parameters associated with the procedure are outlined elsewhere [7]. To summarize, the inlet temperature was 220 °C, aspirator set at 100%, pump rate set at 5%, and the flow rate set at 22%. It is important to note that when preparing the mannitol, lactose, and leucine solution, it was important to add the lactose before the addition of mannitol and leucine to allow the solutes to dissolve easily and properly.

Carrier solutions with different ratios of mannitol:lactose (0:1, 1:3, 1:1, 3:1, and 1:0) were prepared. To each solution, a certain amount of leucine was added to give 5% w/w of the total mass of sugar added to the solutions. Carriers and leucine were dissolved in deionized (DI) water while heating the solution to 75 °C under stirring at 120 rpm. The final solutions were spray-dried under the conditions mentioned elsewhere [7].

### Particle size analysis and sieving

Laser diffraction particle size analyzer (Sympatech, Germany) was used to measure the particle size distribution of salbutamol sulfate and spray-dried carriers. As for final formulations, a specific particle size range was needed; therefore, mechanical sieving was used to separate size fractions of 63–90 μm for spray-dried samples. The collection of particles within 63–90 μm was completed using a Retsch AS 200 Digit Analytical Sieve Shaker (Hoan, Germany) where the collection pan was placed at the bottom which was then followed by the 63-μm sieving pan and finishing with the 90-μm sieving pan; the particles were placed on top of the 90-μm sieving pan where sieving was performed for 30 min with an amplitude of 100 for each of the carriers prior to particle analysis. Particles which fell within the range of 63–90 μm were collected, sealed, and stored in glass vials in an air-conditioned laboratory with a set temperature of 20 °C and relative humidity (RH) of 50% for future use within this study.

### Preparation of DPI formulations

Using the stored 63–90 μm sieved carriers, salbutamol sulfate (SS; 1–6 μm) was introduced such that a final ratio (carrier:SS) of 67.5:1 was obtained. This, then, corresponded to a theoretical dosage of 482 + 1.5 μg of SS per single unit from 1.35 g of each carrier and 20 mg of SS. Mixing was carried out with the use of a Turbula Type T2F (Junkermattstrasse, Switzerland) where each of the formulations became subjected to 30 min of blending at a speed of 72 rpm to ensure a homogeneous formulation.

Each capsule (gelatin, size 3) was filled with 33.22 + 0.1 mg of each formulation where each in vitro deposition used ten (*n* = 10) actuations; each deposition was done in triplicate. Once capsule filling was completed, they were stored for 24 h to decrease the electrostatic charge prior to them being used in the in vitro aerosolization study; all actuations used a dry powder inhaler.

### Differential scanning calorimetry analysis

Perkin Elmer’s (Shelton, Connecticut, USA) Differential Scanning Calorimetry (DSC) 4000 equipped with a Standard Single-Furnace was used to perform thermodynamic analysis; viewing and analyzing the data was completed with the accompanied Pyris Series software. Using a modified protocol outlined by Molina et al. [7], the scanning rate was decreased to 5 °C/min as such rate is known to provide a more thorough thermal analysis [20, 25]. Nonetheless, each of the samples was accurately weighed, where the mass ranged from 4 to 5 mg per sample, on aluminum pans and sealed with an aluminum cap; calibration was completed through the use of indium and zinc prior to any analysis.

### X-ray powder diffraction

To assess the solid state of the engineered particles, X-ray powder diffraction (XRPD) was used. Patterns were collected with Siemens’ Diffraktometer D5000 (Munich, Germany) containing a slit detector copper (Cu) К_α_ source at 30 mA and 40 kV. Carriers were put on a holder (~ 200 mg), leveled, and placed at an angle from the X-ray beam. Data were collected from 5° to 50° on the 2*θ* plane at room temperature (20 °C) with a step rate of 0.1 increments per second.

### Scanning electron microscopy

Shape and surface morphology of all engineered carriers was completed with a JMS-820 scanning microscope (Freising, Germany) where each carrier was placed on a double-sided carbon disk and sputter-coated using Agar Scientific’s S150 Sputter Coater (Essex, UK) with gold (Au) prior to imaging. Sputter coating was performed at 20 mA for 5 min in an argon environment. The type of detector was a secondary electron detector. Scanning electron microscopy (SEM) has operated a voltage of 4 kV and different magnifications were implanted such that an image showed quality resolution.

### In vitro aerosolization study

A multi-stage liquid impinger (MSLI), equipped with a United States Pharmacopeia (USP) stainless steel induction port (Copley Scientific in Nottingham, UK), was used alongside a Critical Flow Controller (Copley TPK) and a High Capacity Pump (Copley HCP5) to study the aerosolization performance of the samples obtained in this research. Prior to commencing, each of the stages in the MSLI was filled with 50 mL of DI water and, following the completion of each individual study, rinsed with another 50 mL of DI water.

Moreover, Eq. 1 was employed to determine the test flow duration, in seconds, used within each deposition to adhere with the USP-specific standard test methods for aerosols, nasal sprays, metered-dose inhalers (MDIs), and dry powder inhalers [26, 27].

1 $$ T=\frac{240}{Q_{\mathrm{out}}} $$

where *Q*_out_ is the volume of air passing through the airflow meter. Testing the airflow through the device was done with a calibrated Test Flow Meter DFM3 (Nottingham, UK) ensuring a 4 kPa pressure drop across the whole device; the Test Flow Meter DFM3 also conforms with USP 33 and Ph. Eur. 6.0.

Each filled capsule was placed inside the device (Cyclohaler®) for actuation. All of the formulations were done a total of three times, equivalent to 30 capsules per formulation. In addition, specific parameters were employed for the analysis of the aerosolization of said capsules including the recovery dose (RD), emitted dose (ED), percent recovery, percent emission, impaction loss, mass median aerodynamic diameter (MMAD), geometric standard deviation (GSD), fine particle fraction (FPF), fine particle dose (FPD), drug loss (DL), dispersibility (DS), and effective inhalation index (EI).

Moreover, RD is defined as the amount of drug (in μg) recovered from the inhaler (I), induction port (IP), mouthpiece (M), and stages 1–5 (S1–5), ED as the amount of drug (in μg) recovered from IP and S1–5, percent recovery as the ratio of RD to the theoretical dose (447 + 1 μg), percent emission as the ratio of ED to RD, impaction loss as the mass fraction of drug in IP and S1 to RD (IP + S1: RD), MMAD as the equivalent aerodynamic diameter at 50% cumulative mass of particles while using a log-probability plot, GSD as the ratio of aerodynamic diameters at 84% and 50% cumulative mass, FPF as the ratio between FPD to RD (FPD:RD), FPD as the sum of drug (in μg) from S3–5, DL as the ratio of the amount of salbutamol sulfate recovered from capsules, mouthpiece, and inhaler to RD [(capsules + (I + M)): RD], and DS as the ratio of FPD to ED (FPD:ED).

Determining the EI of each of the formulations was done as outlined in Molina et al. [7]. All the in vitro aerosolization studies were conducted in an air-conditioned laboratory where the temperature was 20 °C and the relative humidity (RH) was at 50%.

### Determination of salbutamol sulfate in the formulations

The uniformity of salbutamol sulfate in each of the formulations was determined and compared to the theoretical dose of 482 ± 1.5 μg (100% salbutamol sulfate). Ten different samples were taken from each of the formulations in an ordered fashion: eight out of the ten simulating a circle, while the ninth and tenth samples being taken directly from the middle. Carefully weighing the ten samples from each formulation, which yielded a mass range of 10–12 mg, they were introduced to 100 mL of DI water in preparation for HPLC.

### High-performance liquid chromatography

Qualitative and quantitative analysis of salbutamol sulfate was completed by using the protocol published elsewhere [7]. Execution of high-performance liquid chromatography (HPLC) was completed via the Agilent 1100 Series HPLC System (Santa Clara, CA, USA) where a degasser (G1322A), binary pump (G1312A), variable wavelength detector (VWD G1314A), column thermostat (G1316A), and thermostat autosampler (ALS G1329A) were coupled with the Waters Spherisorb 5 μm ODS2 4.6 × 150 mm Analytical Column (Milford, MA, USA); to analyze and view the chromatographs, *ChemStation* Software was utilized. Likewise, internal standards of varying salbutamol sulfate concentrations (0.0, 0.5, 2.5, and 5.0 μg/mL, respectively) were used to calibrate and normalize the results.

### Statistical analysis (ANOVA)

One-way analysis of variance (ANOVA) was used to evaluate the results in this study where statistical probability (*P*) values less than 0.05 were considered a significant difference. The test was followed by Tukey’s honestly significant difference (HSD) test. All data are expressed as the mean + standard deviation where *n* ≥ 3.

## Results and discussion

### Particle analysis and morphology

It has been shown that particle interactions are of great importance in dry powder inhaler (DPI) formulations where the dispersion of the active pharmaceutical ingredient particles from carrier particles is critical for lung deposition [28]. The obtained spray-dried carriers were sieved to get particle size fractions between 63 and 90 μm to ensure the same size fraction was used for all formulations.

**Fig. 1:**
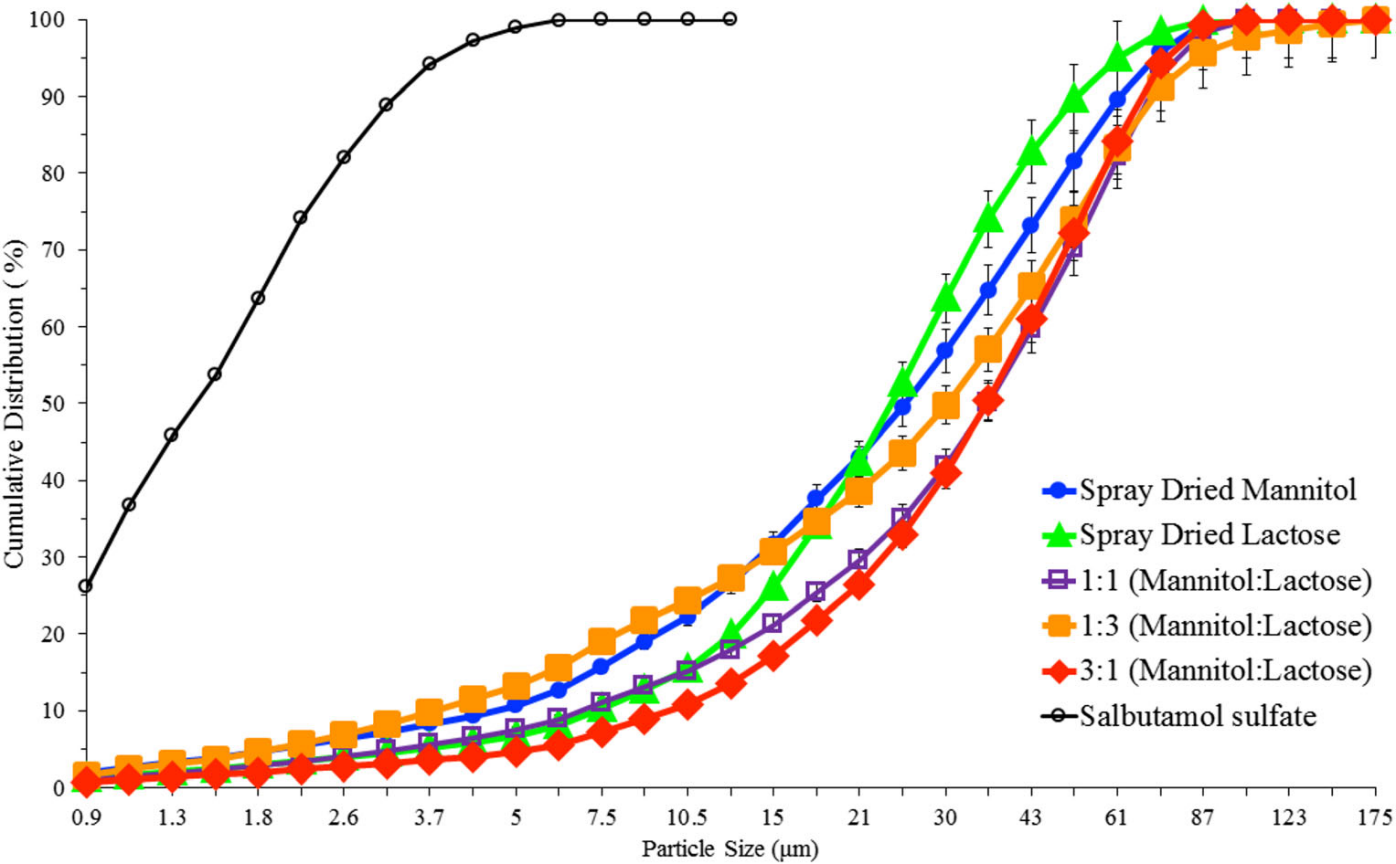
Cumulative particle size distribution of salbutamol sulfate and carriers (*x*-axis is in log scale)

The particle size distribution of SS showed that they are in respirable size (over 99% of particles are less than 6 μm) (Fig. 1). The figure also showed that the spray-dried ternary mixtures exhibited wider distribution (Fig. 1). In order to get 60–93 μm, the obtained samples were sieved and the mentioned fraction was used in DPI formulations.

After sieving (63–90 μm), the amount of powders left on 90 μm sieve was negligible, but around 50% of the co-processed particles lied below 63 μm. This indicates that the spray drying process needs to be optimized to get most of the particles between 63 and 90 μm when considering the scale-up process, but this was out of the scope of the current research. On the basis of the authors’ experience on spray drying of the binary mixtures of lactose:mannitol containing leucine to get larger particles, the feed rate and solid concentration can be increased but the effect of solid concentration on particle size was much higher than the effect of feed rate. In addition, increasing the inlet temperature increased the percentage yield. These parameters should be considered when researchers want to scale-up the materials in the industry. The authors recommend referring to a recent article published in 2019 on scale-up of pharmaceutical spray drying for mechanistic evaluation of parameters when switching from lab scale to large scale [29].

**Fig. 2:**
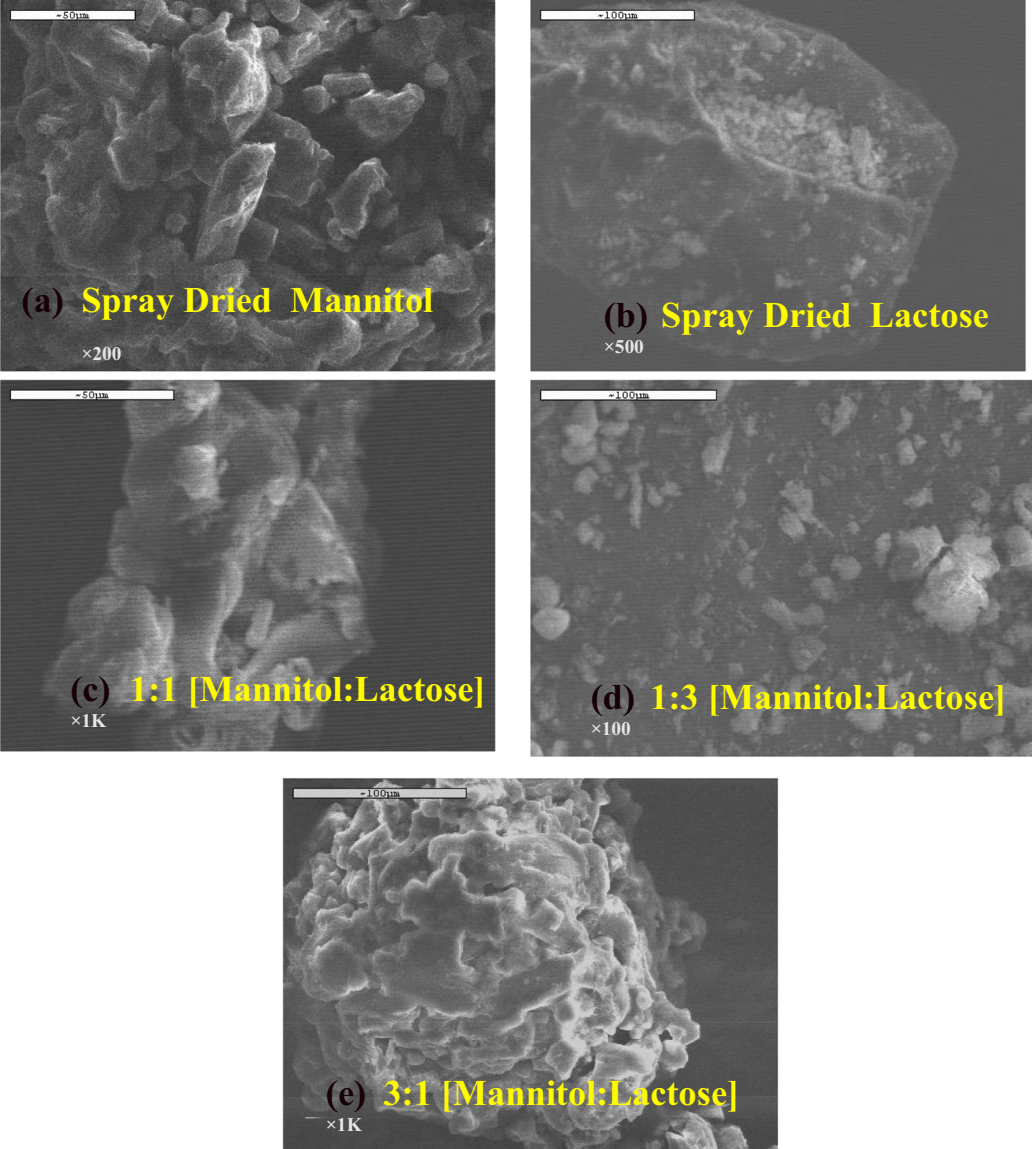
SEM electron micrograms of **a** spray-dried mannitol, **b** spray-dried lactose, **c** SD 1:1 [mannitol:lactose], **d** SD 1:3 [mannitol:lactose], and **e** SD 3:1 [mannitol:lactose] all with 5% leucine (w/w). Different magnifictions were employed to obtain images with resolution

Figure 2 presents the electron micrograms of all the carriers (SD mannitol, SD lactose, different ratios of mannitol:lactose (1:1, 1:3, and 3:1)). Both the SD mannitol and SD lactose carriers were characterized as resembling the structure of a tomahawk while the other ratios of mannitol:lactose (1:1, 1:3, and 3:1) were characterized as the mixtures of spherical particles with some irregular particles. Based on the electron micrograms, it was deduced that some degree of agglomeration took place.

### Solid-state characterization of spray-dried samples

**Fig. 3:**
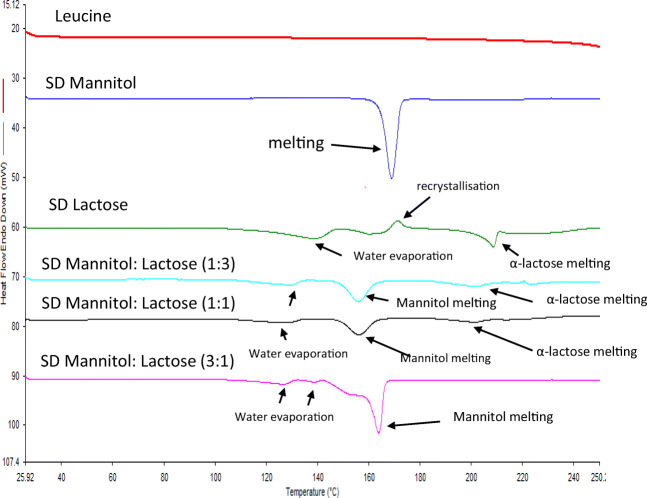
DSC thermal peaks of spray-dried mannitol, spray-dried lactose, and binary mixtures of [mannitol:lactose] at different ratios of 1:3, 1:1, and 3:1 where an exothermic peak points up and an endothermic peak points down (all spray-dried formulations containing 5% leucine)

Figure 3 presents the thermal traces associated with each of the carriers while highlighting the endotherm or exotherm found within each sample. To rule out the possibility of leucine having any thermal events within the 25–250 °C range, a pure sample of leucine was analyzed, and it can be seen from Fig. 3 that leucine had no thermal event within the aforementioned range.

Spray-dried mannitol with 5% leucine (w/w) presented an endothermic peak at 168.79 + 0.35 °C, known to be attributed to the melting of mannitol (α- or β-mannitol) [30, 31] with a resulting enthalpy of 208.66 + 6.87 J/g. Spray-dried lactose with 5% leucine (w/w), however, showed 3 endothermic peaks at 138.88 + 1.06, 160.16 + 0.13, and 208.48 + 0.30 °C, and one exothermic peak at 171.13 + 0.23 °C. The broad first and second endothermic peaks (138.88 + 1.06 °C and 160.16 + 0.13 °C) are attributed to the evaporation of water [32] with an enthalpy of 84.15 + 9.29 and 33.04 + 10.72 J/g respectively, whereas the third endothermic peak (208.48 + 0.30 °C) corresponded to the melting of α-lactose [33] giving an enthalpy of 53.96 + 5.98 J/g. The exothermic peak at 171.13 + 0.23 °C is due to the crystallization of amorphous lactose [33] to α-lactose (Fig. 3). From the spray-dried lactose DSC traces, however, it is difficult to say if this sample is highly amorphous. To confirm if spray-dried lactose is in its amorphous form or if it is only α-lactose, XRPD was used (discussed later). Furthermore, when looking at the carriers containing mixtures of mannitol:lactose with 5% leucine (w/w) and their respective thermal events, it appears that all of the samples experienced temperature shifts compared to spray-dried mannitol or spray-dried lactose containing 5% leucine (Fig. 3). This shift is associated with the introduction of leucine in the samples prior to undergoing spray drying. The 1:3 [mannitol:lactose] carrier experienced three endothermic peaks at 127.22 ± 2.98 (dehydration of water), 156.11 ± 0.16 (mannitol melting), and 201.34 ± 0.08 (α-lactose melting) where the enthalpies for the endothermic peaks were 36.88 ± 3.03, 91.36 ± 9.80, and 33.71 ± 23.48 J/g, respectively. These DSC traces showed that the enthalpy of peaks related to α-lactose is very low which could be an indication of very low contents of the α-anomer in the sample. The absence of the recrystallization peak before the melting peak of α-lactose indicates that the presence of mannitol prevented the conversion of amorphous lactose to crystalline lactose.

When the concentration of lactose was reduced in the samples containing 1:1 [mannitol:lactose], the DSC traces exhibited three endothermic peaks at 129.77 ± 0.00 (water evaporation), 158.19 ± 0.05 (mannitol melting), and 205 ± 3.2 °C (α-lactose melting) with enthalpies of 49.07 ± 11.40, 166.39 ± 19.04, and 18.5 ± 6.2 J/g, respectively.

In the case of the carrier having a high concentration of mannitol in the sample (3:1, mannitol:lactose), the peaks regarding α-lactose disappeared. The first two endothermic events are due to water evaporation and the third peak at 163.94 °C is due to the melting of mannitol with an enthalpy of 178.22 ± 25.63 J/g. The 3:1 [mannitol:lactose] carrier failed to show any traces of α-lactose melting and also the absence of any crystallization peak suggesting that somehow the presence of mannitol in the sample prevents the conversion of amorphous lactose to α-lactose or β-lactose.

**Fig. 4:**
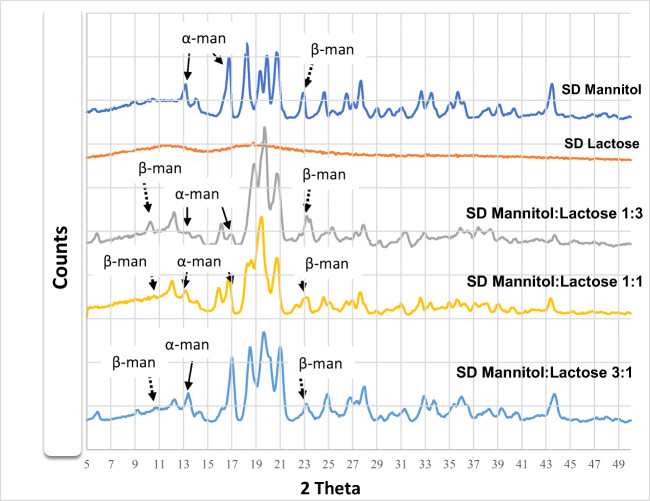
Powder X-ray diffraction patterns of spray-dried mannitol, spray-dried lactose, and different ratios of mannitol:lactose 1:3, 1:1, and 3:1 (all formulations contain 5% w/w leucine)

Figure 4 shows the powder X-ray diffraction (PXRD) peaks associated with each carrier, aiding in the characterization of the carrier’s polymorphic form. PXRD can identify if the sample is crystalline or amorphous. It also provides specific peaks to distinguish between different polymorphic forms of crystalline mannitol (α-, β-, and δ-) and different lactose anomers. PXRD of spray-dried lactose differed remarkably from all the other carriers due to its amorphous nature and confirms the results obtained in Fig. 3 (DSC).

It has been reported that α-mannitol shows peaks at 9.6°, 13.8°, and 17.2°, β-mannitol at 10.56°, 14.7°, and 23.4, and δ-mannitol at 9.74° [21]. With the results presented in Fig. 4, it was determined that all samples containing mannitol were composed of mainly α-mannitol and β-mannitol as shown in Fig. 4 (some of the diagnostic peaks for α- and β-mannitol are shown with arrows).

### In vitro analysis of DPI formulations

#### Deposition of salbutamol sulfate evaluation

**Fig. 5:**
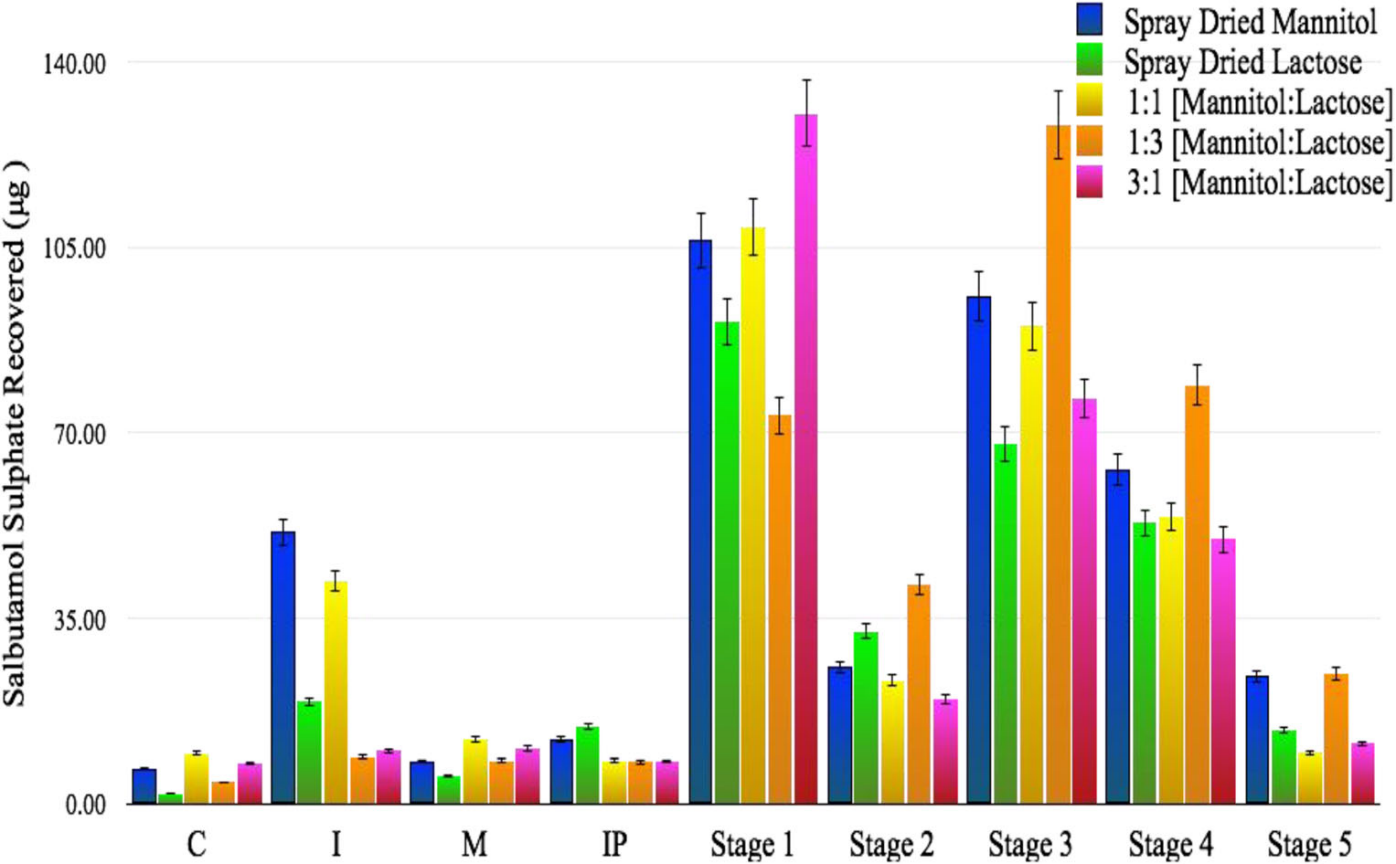
Aerosolization performance of each formulation (spray-dried mannitol, spray-dried lactose, 1:1 [mannitol:lactose], 1:3 [mannitol:lactose], and 3:1 [mannitol:lactose]) highlighting the amount of salbutamol sulfate recovered

Aerosolization performance of all of the formulations is summarized in Fig. 5 where the amount of salbutamol sulfate deposited into each of the stages of the multi-stage liquid impinger (MSLI) is shown [capsules (C), inhaler (I), mouthpiece (M), induction port (IP), stage 1, stage 2, stage 3, stage 4, and stage 5]. The figure clearly shows that the aerosolization behavior of each formulation is different. Figure 5 shows that the amount of salbutamol sulfate deposited in stages 3 and above are much higher than other DPI formulations (stage 3 above is the region for the deposition of particles in the lung). This indicates that spray drying of both lactose and mannitol in a single solution can enhance the deposition of salbutamol sulfate at lower airways of the lungs; to discuss these data in detail, Table 1 was generated.

**Table 1:** Recovered dose (RD), emitted dose (ED), percent recovery, percent emission, percent impact loss, mass median aerodynamic diameter (MMAD), geometric standard deviation (GSD), fine particle dose (FPD), fine particle fraction (FPF), drug loss (DL), dispersibility (DS), and effective inhalation index (EI) of salbutamol sulfate obtained from each of the different formulations (spray-dried mannitol, spray-dried lactose, 1:1 [mannitol:lactose], 1:3 [mannitol:lactose], and 3:1 [mannitol:lactose])

Formulation	RD (μg)	ED (μg)	Recovery (%)	Emission (%)	Impact loss (%)	MMAD (μm)	GSD (μm)	FPD	FPF (%)	DL (%)	DS (%)	EI
Spray-dried mannitol	213.2 + 59.4	147.9 + 53.4	44.3 + 12.3	73.0 + 28.6	28.89 + 3.2	3.0 + 0.1	2.01 + 0.01	75.8 + 43.1	38.1 + 21.8	29.1 + 30.2	48.5 + 14.2	10.32 + 2.63
Spray-dried lactose	297.3 + 175.08	272.8 + 179.6	61.83 + 36.4	88.9 + 7.4	35.9 + 4.0	3.0 + 0.1	2.13 + 0.08	134.8 + 91.4	43.08 + 7.3	12.00 + 8.1	48.26 + 5.3	11.48 + 0.63
M:L (1:1)	348.5 + 68.1	294.2 + 87.4	72.4 + 14.1	83.63 + 14.2	34.4 + 7.7	3.1 + 0.1	2.03 + 0.04	153.8 + 83.9	42.5 + 18.4	18.9 + 14.8	49.30 + 15.6	11.17 + 1.51
M:L (1:3)	371.0 + 144.4	354.0 + 140.7	77.1 + 30.0	95.2 + 0.9	23.29 + 5.8	3.0 + 0.1	2.08 + 0.03	231.6 + 103.2	61.42 + 4.2	5.93 + 1.0	64.4 + 3.7	12.51 + 0.21
M:L (3:1)	316.0 + 38.22	295.6 + 34.9	65.7 + 7.9	93.5 + 0.4	44.6 + 11.7	3.0 + 0.1	2.04 + 0.03	137.6 + 47.1	42.8 + 9.3	8.9 + 1.5	45.8 + 10.1	11.68 + 0.38

Table 1 presents the recovered dose (RD), emitted dose (ED), percent recovery, percent emission, percent impact loss, mass median aerodynamic diameter (MMAD), geometric standard deviation (GSD), fine particle dose (FPD), fine particle fraction (FPF), drug loss (DL), dispersibility (DS), and effective inhalation index (EI) for salbutamol sulfate obtained from each of the different formulations (spray-dried mannitol, spray-dried lactose, 1:1 [mannitol:lactose], 1:3 [mannitol:lactose], and 3:1 [mannitol:lactose]) and correlates with the data already presented in Fig. 5. With that being said, spray-dried mannitol accounted for receiving the least amount of salbutamol sulfate for RD, ED, percent recovery, and percent emission with 213.23 + 59.44 μg, 147.97 + 53.44 μg, 44.33 + 12.36%, and 73.03 + 28.66% respectively; the 1:3 [mannitol:lactose] carrier, however, received the highest amount of salbutamol sulfate for RD, ED, percent recovery, and percent emission with 371.01 + 144.43 μg, 354.07 + 140.71 μg, 77.13 + 30.03%, and 95.21 + 0.95% respectively.

The 1:3 [mannitol:lactose] carrier received the lowest impact loss with 23.29 + 5.83% whereas the highest belonged to the 3:1 [mannitol:lactose] carrier with 44.65 + 11.75%. These results suggest that the 1:3 [mannitol:lactose] carrier obtained the best aerosolization performance among the carriers. With respect to MMAD, the 1:1 [mannitol:lactose] carrier obtained the largest diameter with 3.10 + 0.06 μm, whereas spray-dried lactose obtained the lowest diameter with 3.00 + 0.18 μm. In addition, the 1:1 [mannitol:lactose] carrier also received the lowest GSD with 2.03 + 0.04 μm whereas the highest GSD came from spray-dried lactose with 2.13 + 0.08 μm. With respect to FPD, the 1:3 [mannitol:lactose] carrier obtained the highest amount with 231.63 + 103.21 μg and the lowest amount was from spray-dried mannitol with 75.82 + 43.07 μg. The 1:3 [mannitol:lactose] carrier received the highest FPF with 61.42 + 4.21% whereas the lowest was from spray-dried mannitol with 38.10 + 21.84%. These results prove that the 1:3 [mannitol:lactose] carrier was the best engineered carrier among all the carriers. The 1:3 [mannitol:lactose] carrier also received the lowest drug loss with 5.93 + 1.04% whereas the highest drug loss was from spray-dried mannitol with 29.10 + 30.22%. Moreover, the 1:3 [mannitol:lactose] carrier had the highest dispersibility with 64.49 + 3.77% and effective inhalation index with 12.51 + 0.21 whereas the lowest dispersibility came from the 3:1 [mannitol:lactose] carrier with 45.82 + 10.19% and the lowest effective inhalation index came from spray-dried mannitol with 10.32 + 2.63. Overall, taking all of the measurements as a whole and not individually, the 1:3 [mannitol:lactose] carrier navigated itself as having the best aerosolization performance when compared to the other carriers. That being said, this carrier is the most effective at delivering salbutamol sulfate to the lower respiratory tract via a dry powder inhaler. The table shows that the presence of 25% mannitol in spray-dried formulation significantly can enhance the aerosolization performance compared to when lactose alone was used in the spray-dried formulation (an improvement in FPF from around 43 to 61%). It has already been shown that the presence of magnesium stearate as a third component in DPI formulation can enhance the deposition of orally inhaled drugs and this was discussed in an article published by Shur et al. who pointed out that binary or ternary mixtures in DPI formulations could be important to enhance the quality of DPI formulations for patients [33]. Currently, they are 6 DPI formulations approved by FDA which are in the market. This indicates the importance of the research on binary or ternary mixtures of formulations in DPI field to enhance the quality of orally inhaled formulations.

### Assay of salbutamol sulfate in the samples

**Fig. 6:**
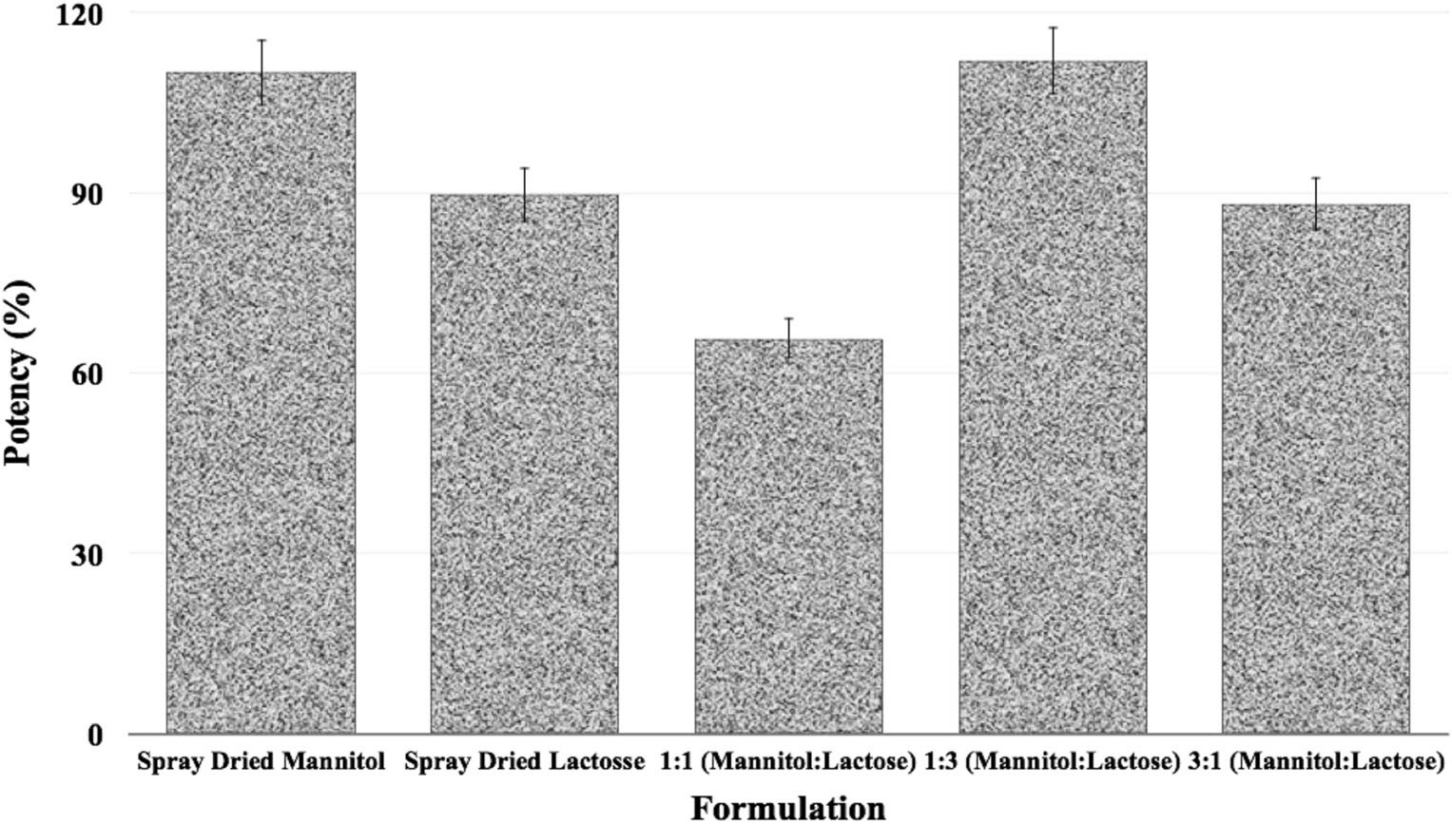
Percent potency of salbutamol sulfate in each formulation: spray-dried mannitol, spray-dried lactose, and different ratios of mannitol:lactose 1:3, 1:1, and 3:1 (all formulations contain 5% w/w leucine)

The amount of salbutamol sulfate in each formulation was assayed using HPLC method mentioned earlier in the manuscript (“High-performance liquid chromatography” section). Figure 6 shows the percentage of the drug in each formulation (the theoretical value should be 100%). With respect to their potency, all of the formulations adhered to the required specification set by the US Food and Drug Administration (75–125% of the nominal dose) except the 1:1 formulation showed 66% salbutamol sulfate which indicates some of the drug particles did not attach to the carrier surfaces and were easily lost during discharge or adhered to the wall of glass vial. The potency of the rest of the formulations for salbutamol sulfate was in the range of 112–88% (Fig. 6). These results indicate that the formulation that contained 3:1 [mannitol:lactose] carrier had the best content homogeneity among all of the carriers where the potency was around 88% with the least CV of 0.75%; the coefficient of variation was a measure of content homogeneity.

The results of the current study showed binary mixtures (in the case of considering leucine as a carrier it could be ternary mixtures) of spray-dried sugars containing leucine can improve the aerosolization performance of DPI formulation. This is ongoing research and the authors are planning to do a mechanistic study on various sugars that can be used in DPI formulations containing orally inhaled drugs with different physicochemical properties.

Moreover, obtaining qualitative and quantitative data via analytical techniques, like that of AFM, BET, and NMR, and determining the potential and kinetic surface energies of the engineered carriers will provide answers that will allow for the emergence of innovative research questions.

## Conclusion

The results presented in this study have proven that spray drying of binary mixtures of mannitol-lactose containing leucine are in favor of enhancing the aerosolization performance of salbutamol sulfate compared to when the single carrier solution was spray-dried. These results also showed that among binary mixtures of mannitol:lactose, the aerosolization performance of the 1:3 [mannitol:lactose] carrier was successfully achieved, and this achievement was measured in the carrier’s FPF which was 61.42 + 4.21%. Solid-state analysis proved that the spray-dried lactose was in its amorphous polymorphic form whereas spray-dried mannitol showed crystalline structure containing α- and β-polymorphic forms when the concentration of mannitol was low. Moreover, this was not the case when binary mixtures of mannitol-lactose were spray-dried where the concentration of mannitol was high. This research opens up the idea of using binary mixtures of carriers in spray-dried formulations to enhance the aerosolization performance of drugs in DPI formulations.
